# Circadian rhythms of European and African-Americans after a large delay of sleep as in jet lag and night work

**DOI:** 10.1038/srep36716

**Published:** 2016-11-07

**Authors:** Charmane I. Eastman, Victoria A. Tomaka, Stephanie J. Crowley

**Affiliations:** 1 Biological Rhythms Research Laboratory Department of Behavioural Sciences, Rush University Medical Center Chicago, Illinois, 60612, USA

## Abstract

Jet travel and night shift work produce large changes in when people sleep, work and eat; a challenge that was not encountered during most of our evolution. Successful adaptation would require the internal, master, circadian clock to make large phase shifts to reduce the circadian misalignment between circadian rhythms and the times for sleep, work and meals. We compared African-Americans and non-Hispanic European-Americans in how much their circadian clocks shifted after a 9 hour phase delay of the light/dark, sleep/wake and meal schedule, which has similarities to flying west or sleeping in the daytime after night shifts. We also measured their free-running circadian periods using a forced desynchrony protocol with a 5-h day. European-Americans had longer free-running periods and larger phase delays than African-Americans. Correlations (among all subjects, just African-Americans and just European-Americans) showed that longer circadian periods were associated with larger phase delays. Larger phase delays, facilitated by longer circadian periods, reduce jet lag after westward travel and make it easier to work night shifts and sleep during the daytime after night work. On the other hand, a shorter circadian period, which makes one more of a morning-type person, is better for most people given our early-bird dominated society.

With the advent of electricity and jet travel, the modern circadian rhythm ailments of jet lag from travel across time zones and shift work disorder due to night work were created. Jet travel and night work result in a large change in the time for sleep and wake, a large phase shift, producing circadian misalignment between sleep, work, meals and the internal circadian rhythms. Circadian misalignment leads to a myriad of health, safety and productivity problems^1^,^2^,^3^,^4^,^5^,^6^,^7^,^8^,^9^,^10^,^11^,^12^,^13^,^14^.

Our lab has focused on developing techniques to phase shift circadian rhythms using appropriately timed light, dark (sleep) and melatonin. Jet lag can be reduced or eliminated by phase-shifting circadian rhythms to the new time zone before the flight and/or speeding up the phase shifting of circadian rhythms after the flight^7^,^9^. Night work could be made safer and healthier and daytime sleep could be lengthened and improved by shifting circadian rhythms to align, at least partially, with daytime sleep^8^,^15^. In two laboratory studies we carefully timed bright light to advance (shift earlier in time)^16^ or delay (shift later in time)^17^ circadian rhythms, by determining the circadian phase of each individual so that the bright light could be applied at the same circadian phase in all subjects. We reasoned that this would reduce the large individual differences in the magnitude of the phase shifts that are typically seen in this type of study. However, large individual differences remained. A colleague suggested looking at race/ethnicity as a source of these individual differences^18^. We did and were surprised to see differences between Blacks/African-Americans and Whites/Hispanics in the magnitude of phase shifts, despite having very few Black subjects in these samples. It was not that one group shifted more than another, but rather that African-Americans phase *advanced* more whereas Whites appeared to phase *delay* more^19^. An explanation could be that the endogenous circadian period (also called the free-running or intrinsic period (τ)) was shorter in African-Americans. This prompted our analysis of the circadian period from data being gathered as part of ongoing studies designed to generate phase response curves (PRCs)^20^,^21^,^22^. That analysis^19^ revealed a shorter free-running circadian period (τ) in African-Americans. When all the PRC studies were completed, producing larger samples, the τ for the 20 African-Americans was significantly shorter than for the 55 Whites (p < 0.001)^23^.

We pursued this line of investigation with two studies that were intentionally designed to compare African-Americans and non-Hispanic European-Americans in two fundamental circadian rhythm properties: 1) the free-running circadian period, and 2) the response of the circadian system to a large change in the timing of zeitgebers. Zeitgebers are time cues that are capable of entraining (synchronizing) free-running circadian rhythms to the 24-h day, and the light-dark (LD) cycle is the most powerful zeitgeber. In one study, we advanced the time of the LD cycle, sleep and meals 9 h^24^, and in the other, reported here, we delayed these factors 9 h (please see Fig. 1). The advance of 9 h is similar to flying east across 9 time zones, and the delay is similar to flying west. For convenience, we call the first study the Kenya study and the second the Japan study, because Kenya is 9 time zones east of our lab in Chicago and Japan is 9 time zones west. Both studies were identical up to and including the third phase assessment (see Fig. 1 in both studies), after which the LD cycle, sleep and meal schedules were advanced (Kenya) or delayed (Japan). All the figures and tables in the current study (Japan) correspond to the figures and tables in our previous study (Kenya) for ease of comparison.

In both studies, we collected participant-reported race of parents and grandparents and buccal DNA samples to estimate genetic ancestry. We do not know of any other studies besides ours which examined the free-running circadian period or circadian rhythm phase shifts after shifts of zeitgebers in people of different race/ethnicity/ancestry.

## Results

Table 1 shows that the % European ancestry and the % Sub-Saharan African ancestry was very different in the African- Americans compared to the European-Americans, as expected. For the African- Americans, the average Sub-Saharan African ancestry was 79% and ranged from 60 to 96%, and the average European ancestry was 13% and ranged from 0 to 35%. For the European-Americans, the average European ancestry was 86% and ranged from 67 to 100%, and the average Sub-Saharan African ancestry was 6% and ranged from 0 to 21%. There were no differences between African and European-Americans in % East Asian or % Indigenous American.

Table 1 also shows that there were more morning-types among the African- Americans, and the difference in the average morningness-eveningness score (MEQ) was statistically significant (p < 0.05). The mid sleep time on free days (MSF) was earlier for the African- Americans, but this difference did not reach statistical significance. There were no significant differences between African and European-Americans in age or BMI (Table 1).

### Free-Running Circadian Period (τ)

The average free-running period (τ) of the 45 subjects was 24.19 ± 0.24 h and ranged from 23.66 to 24.75 h. There was overlap in the distributions of circadian periods for African and European-Americans (Fig. 2), but the difference between the groups was statistically significant [t = 3.884, p < 0.001] (Table 2). Seven of the 23 African-Americans (30%), and two of the 22 European-Americans (9%, both women) had circadian periods <24.00 h. This difference in the number of τs < 24 h between African and European-Americans was not significant by a 2-sided Chi-Square test (p = 0.074).

There were no sex differences in circadian period for the African-Americans (men 24.07 ± .23, N = 10; women 24.04 ± 0.17, N = 13). For European-Americans, women had a descriptively shorter circadian period than men (24.20 ± 0.29, N = 10 vs 24.39 ± 0.16 h, N = 12), which was a trend by a 2-tailed t-test (p = 0.065).

### Phase Angle of Entrainment during Baseline

The phase angle of entrainment – the temporal relation between the internal, master, circadian clock and external zeitgebers – was associated with the endogenous free-running circadian period (Fig. 3). Subjects with shorter circadian periods had earlier circadian rhythms (earlier dim light melatonin onsets (DLMOs)) relative to dark (relative to bedtime = lights out), and subjects with longer circadian periods had later circadian rhythms relative to dark (r = + 0.67, p < 0.001 for the African-Americans (N = 23); r = + 0.51, p = 0.016 for the European-Americans (N = 22); r = + 0.56, p < 0.0001 for both (N = 45)). The average phase angle was larger for the African-Americans (they had earlier DLMOs relative to dark) than for the European-Americans (−2.3 ± 1.5 h compared to −1.9 ± 1.5 h) but this difference was not statistically significant by t-test (Table 2).

### Phase Shifts to 9-hour Phase Delay of Zeitgebers

The phase shifts of the master circadian clocks (marked by the DLMOs) after the 9-h delay of Zeitgebers are shown in Fig. 4. There was much overlap between the African and European-Americans in the magnitude of the phase delay, but on average the European-Americans delayed more (Table 2 and Fig. 4). The smallest phase delay for the European-Americans was about 2 h whereas 7 African-Americans had smaller phase delays than this, and 4 had phase delays <1 h. Assuming a linear delay from the third to the fourth phase assessments (over 4 days), on average the European-Americans delayed by 54 min/day, whereas the African-Americans delayed by 36 min/day.

The phase shift of the master circadian clock due to the 9-h delay of zeitgebers depended on the free-running circadian period (Fig. 5). Subjects with longer circadian periods had larger phase delays of the DLMO (r = −0.60, p = 0.003 for the European-Americans; r = −0.52, p = 0.011 for the African-Americans; and r = −0.66, p < 0.0001 for both).

### Correlations with Ancestry Estimates

When the European-Americans and African-Americans were combined (N = 45), a positive correlation was seen between circadian period and European ancestry (r = + 0.47, p < 0.01, 2-tailed) and a negative correlation was seen between circadian period and Sub-Saharan African ancestry (r = −0.46, p < 0.01). When the groups were considered separately, these correlations between and ancestry estimates were small and not significant. When the European-Americans and African-Americans were combined (N = 45), a negative correlation was seen between phase shift and European ancestry; larger phase delays with greater % European (r = −0.46, p < 0.01, 2-tailed) and a positive correlation was seen between phase shift and Sub-Saharan African ancestry (r = +0.47, p < 0.01). But again, when the groups were considered separately, these correlations between phase shift and ancestry estimates were small and not significant.

## Discussion

In this study, we delayed the light/dark sleep/wake and meal schedule by 9 h, similar to flying west across 9 time zones, like from Chicago to Japan. After 3 days and nights living in our laboratory on Japan time, the European-Americans had delayed more than the African-Americans. There was a large overlap in the distributions of phase shifts between the African and European-Americans, and large individual differences in the magnitude of the phase shifts in both groups (Fig. 4). Nevertheless, the difference between groups was highly significant. To put the difference in perspective, if the number of days living in our laboratory on Japan time was extended (a very costly endeavor) and the rate of phase shift remained the same, then it would take 10 days (on average) for the European-Americans to phase delay 9 h, but 15 days for the African-Americans to delay that far.

The factor we measured that best explained the individual differences in phase shift magnitude was the free-running, intrinsic, circadian period (τ). In the current study, the Japan study, we found that African-Americans had shorter endogenous free-running circadian periods than European-Americans (24.06 h compared to 24.31 h, Fig. 2 and Table 2), confirming the results of our previous study, the Kenya study^24^, (24.07 h compared to 24.36 h). In the current study, there was a significant correlation between phase shifts and circadian period (r = −0.66, Fig. 5); subjects with longer periods had larger phase delay shifts. This was similar to the finding from our previous study, the Kenya study (r = −0.56). To our knowledge, no other published data exist besides ours correlating phase shifts after a shift of zeitgebers with circadian period for individuals within a single species. To understand the correlations we found, imagine a person with a free-running period of 24.5 h, whose circadian clock drifts later by 0.5 h/day. If the 9 h delay of zeitgebers that we imposed pushed the clock later by 1.5 h/day, then the total delay would be 2 h/day. On the other hand, imagine a person with a free-running period of 23.5 h, whose circadian clock drifts earlier by 0.5 h/day. If the zeitgebers also pushed the clock later by 1.5 h/day, then the total delay would only be 1 h/day. In other words, a longer circadian period helps the individual to delay more.

The differences in circadian period in the current study between African-Americans and European-Americans (24.06 h compared to 24.31 h, a 15 min difference) may seem trivial to the casual reader, but a small difference in circadian period can cause a much larger difference in phase shift or the phase angle during entrainment (see Figs 3 and 5). Micic *et al.*^25^ found a 12 min difference in circadian period between people with the delayed sleep-wake phase disorder (DSWPD) and normally sleeping controls. The DSWPD subjects had longer circadian periods, scored more towards eveningness on the MEQ, had later DLMOs, and fell asleep and woke up later on free days. We can attribute much of these differences to the difference in circadian period.

In this study (Japan) the circadian rhythms of all the subjects delayed after the 9 h delay of zeitgebers. In our previous study (Kenya)^24^ several subjects delayed after the 9 h *advance* of zeitgebers, which is called antidromic re-entrainment or, simply put, phase shifting in the wrong direction (compare Fig. 4 in both papers). Antidromic re-entrainment is common after large, abrupt phase advances of zeitgebers^26^,^27^,^28^,^29^. European-Americans were more likely to phase shift in the wrong direction^24^, which makes sense given their longer free-running circadian periods. We expected the African-Americans to advance more after the 9 h advance of zeitgebers (the Kenya study^24^), because of their shorter circadian periods, but this was not the case. Figure 4 of that study shows that if we compare only the subjects who advanced, the African-Americans clearly did not advance more than the European-Americans. Furthermore, if we compare only the subjects who delayed, the European-Americans delayed more. Figure 4 from the Kenya study also shows that the largest phase shifts (in either direction) were made by the European-Americans. This difference was confirmed by a significant t-test between the average absolute phase shifts (2.5 h for the European-Americans and 1.4 h for the African-Americans). These results suggest that the jet lag of African-Americans after real flights will last longer regardless of whether they fly east or west. Thus, measures to prevent or reduce jet lag^7^,^9^ may be even more important for African-Americans.

Of course, complete re-entrainment to a new time zone, a phase delay of 9 h in this case, is not necessary to overcome jet lag. We suggest that jet lag will be minimal as long as the body temperature minimum (Tmin), the sleepiest time of day which ordinarily occurs during sleep, delays far enough to fall into the beginning of the new, delayed, sleep episodes^7^,^9^. A phase delay of this amount will permit adequate sleep because it will push the circadian rise of temperature and alertness later, preventing the early awakenings typically seen after flying west. Furthermore, alertness will be maintained during the day until late in the evening when approaching the time of the Tmin. The larger the phase delay, the sooner jet lag symptoms will disappear.

Circadian misalignment from jet travel across time zones usually dissipates in a few days, and there are methods to prevent or reduce it with the proper timing of light, dark and melatonin^7^,^9^. Night shift work, however, produces extreme, debilitating and chronic circadian misalignment between the times for sleep, work, meals and the internal circadian rhythms resulting in numerous health and safety problems. Night shift workers include police, guards, nurses, healthcare support staff, miners and factory workers^30^. African-Americans are more likely to have jobs that require shift work^30^,^31^, and thus their exposure to shift work and its health consequences is greater.

It is not possible to reduce circadian misalignment with rapidly rotating shift work schedules, because circadian rhythms cannot phase shift fast enough to keep up with the changes in the times for work and sleep^8^,^15^. Even the circadian rhythms of most permanent (fixed) night shift workers do not delay enough to align with night work and day sleep, because workers usually revert to sleeping at night (at conventional times) on days off and because they are usually exposed to bright advancing morning light on the way home from work. Our simulated night shift studies^8^,^32^,^33^ with permanent night work schedules have shown that a partial delay of circadian rhythms, moving the Tmin, the sleepiest time, out of the night shift and into daytime sleep, can be achieved with the use of short episodes of intermittent bright light during night shifts, dark sunglasses on the way home from work and late sleep schedules on days off. The current study showed that African-Americans had smaller phase delays than European-Americans, which means they are less likely to delay their circadian rhythms enough to reduce circadian misalignment when working nights and sleeping during the day. When promoting interventions to help night workers reduce circadian misalignment, we should emphasize that these methods are especially important for African-Americans.

Research is needed to confirm that the techniques to increase phase shifts and reduce circadian misalignment from jet travel and night work (timed light, dark and melatonin) work well for African-Americans, and how best to increase phase advance and phase delay shifts in African-Americans.

It has long been known that the phase relationship that the circadian clock assumes within the 24-hour day (called the phase angle of entrainment) depends on the free-running circadian period^34^,^35^. This has more recently been shown in monkeys^36^ and humans^37^,^38^. In the current study (Japan), we found a correlation between the phase angle of entrainment and the free-running circadian period (r = +0.56, Fig. 3), replicating the correlation we found in the Kenya study^24^ (r = +0.52). These correlations show that the shorter the circadian period, the earlier circadian rhythms occur when entrained to the 24-h zeitgebers. In other words, shorter circadian periods tend to make people morning-types or early birds. In line with this, we found that African-Americans scored slightly, but significantly, higher on morningness compared to European-Americans (see MEQ score, Table 1). African-Americans also had slightly earlier baseline DLMOs, sleep schedules and phase angles (Table 2), but none of these differences were statistically significant. Malone *et al.*^39^ used data from the UK Biobank study with thousands of subjects and found that morning versus intermediate type was 1.4 times more prevalent in Black/Black British than Whites.

Humans evolved in East Africa around the equator. Some people migrated into West Africa, and some migrated north and eventually into Europe. The people who lived in Europe had to adapt to a photoperiod (duration of daylight) that changes with seasons. We speculate that natural selection over thousands of years resulted in longer circadian periods for humans that lived in northern latitudes. Pittendrigh & Daan^35^ showed that a period longer than 24 h, which is characteristic of diurnal animals, helps them keep an appropriate phase angle relative to dawn as the photoperiod changes. Selection pressure to change circadian period to values slightly away from 24 h at higher latitudes has been extensively discussed^40^,^41^ Presumably, there was no selection pressure to change circadian period for the humans who migrated into western Africa because they were still living around the equator where the photoperiod is relatively constant throughout the year. The “resonance hypothesis” states that a circadian period closest to 24 h confers fitness, perhaps because it requires less phase shifting of the circadian clock for entrainment^40^,^42^,^43^. It is easy to understand natural selection for lighter skin at higher latitudes to get more sunlight for producing vitamin D^44^,^45^, and a longer circadian period to adapt to a seasonally changing photoperiod. This does not mean that other human adaptations, such as intelligence or creativity, differed by latitude given the great challenges of life during our early evolution.

The United States of America is a nation of immigrants who arrived recently, in evolutionary terms. Many Europeans came to the USA by boat, especially in the 1800s, presumably with the longer circadian period that they inherited from their ancestors. Africans from western Africa around the equator also came to the USA by boat, but unfortunately not by choice. Instead they were sold into slavery to work on American southern plantations starting in the 1500s^46^,^47^, it was not outlawed until 1865. Most African-Americans are descendents of these people, and we speculate that they arrived in America with the original, shorter, African circadian period.

A longer circadian period might have been adaptive for keeping up with seasonal changes in daylight before the widespread use of electricity that essentially gives us a fairly stable summer-like photoperiod year round. Currently, however, the challenges of an industrialized and mostly urban society are different. For most people it is better to have the original shorter African circadian period, because it should help to produce an earlier sleep schedule in this early bird dominated world, avoiding the perils of social jet lag^48^,^49^. It should also make people less likely to develop DSWPD. For permanent night workers or those flying west across time zones, however, a longer circadian period would be better.

## Methods

The methods were the same as for our previous study (Kenya)^24^ except that the protocol had a delay of sleep in the last few days (11–13) rather than an advance.

### Ethical Approval

The study was approved by the Rush University Medical Center Institutional Review Board and conformed to the standards set by the Declaration of Helsinki. Written informed consent was obtained from all subjects prior to their participation. Subjects were reimbursed for their participation.

### Subjects

We enrolled 53 subjects 5 to 6 days before the start of the 14-day laboratory study. Of these, 47 started the study, 46 completed the study and 45 could be classified as African-American or European-American and are included in Table 1.

Subjects completed our Family/Ancestor Questionnaire and were asked to check all of the following categories that applied to them: White, Black or African-American, Asian, Hispanic or Latino, European, Middle Eastern, Far East Asian, Southeast Asian, Indian Subcontinent, North African, Afro-Caribbean, American Indian or Alaska native, Native Hawaiian or other Pacific Islander, Other, Don’t Know. A short description was provided for each category. Subjects did the same for their biological mother, biological father, and their four grandparents. All the European-Americans in Table 1 endorsed White for all 6 of their relatives except for one subject who checked “Don’t Know” for one grandparent. All of the African-Americans in Table 1 endorsed Black/African-American for all 6 of their relatives except for 6 subjects: 3 checked “Don’t Know” for two grandparents, one checked “Other” for one grandparent, one checked Indian Subcontinent for one grandparent and one checked “Afro-Caribbean” for father and two grandparents. None of the subjects checked “Hispanic or Latino” for any of their 6 relatives.

Buccal (cheek) swabs were used to collect a DNA sample from each subject during the study, and were processed by Ancestry*by*DNA, DNA Diagnostics Center, Fairfield, OH. This company performed biogeographical ancestry estimates based on ancestry informative markers, also known as population-specific alleles, which show large frequency differences between populations^50^,^51^. Results were returned several weeks later with percents for each subject in 4 categories: European, Sub-Saharan African, East Asian and Indigenous American (Table 1). The results section showed that there were weak, but statistically significant, correlations between ancestry (% European or % Sub-Saharan African) and circadian period and between ancestry and phase shift when all the subjects were combined, but when the groups were considered separately the correlations were very small and not significant. Thus, these ancestry estimates were not useful for predicting circadian variables within a race. The value in obtaining these DNA samples appears to be limited to making sure we categorized people into the correct groups. For example, in the current study one subject who completed the study listed both parents and all grandparents as being Black/African-American. The results of his genetic ancestry, however, indicated that he was 56% European and 30% Sub-Saharan African, so we could not include him in either the European-American or African-American group. A similar situation occurred for one self-identified African-American subject in the Kenya study^24^ who was 49% European and 46% Sub-Saharan African. We didn’t include their data because we want our results to generalize to the majority of African-Americans. This careful selection of subjects for the groups had not been done before our studies, and shows the benefit of confirming ancestry with DNA testing.

Subjects were young, mostly in their 20 s and 30 s, and healthy. They were not taking any prescription medications except for 6 women on oral contraceptives (3 African-Americans and 3 European-Americans). Due to the length of the study (14 days in the lab) most subjects were unemployed. Subjects were screened by telephone followed by an in-person interview and several questionnaires. Exclusion criteria included body mass index (BMI) >35 kg/m^2^, night shift work in the preceding month, smoking and excessive alcohol or caffeine consumption. Subjects were given urine tests for common drugs of abuse and nicotine, and were breathalyzed 5 to 6 days before starting the study and on days 1 and 7 of the study. Subjects completed the Munich Chronotype Questionnaire (MCTQ)^52^ and the Owl-Lark (Morningness-Eveningness) Questionnaire (MEQ)^53^ during the study (Table 1).

### Protocol

This study took place in the Biological Rhythms Research Laboratory in Chicago from November 2014 to July 2016. Subjects were run in groups of three, and there was usually a mixture of African and European-Americans in each group.

**Figure 1:**
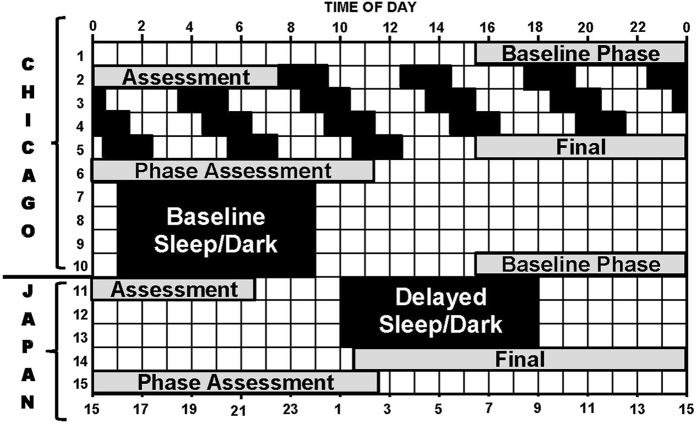
Protocol diagram. The dim light melatonin onset (DLMO) was determined from 30 min saliva samples obtained during the circadian phase assessments (days 1–2, 5–6, 10–11 and 14–15), and was used as a marker for the phase of the master circadian clock. Subjects were kept in temporal isolation and put on a 5-h ultradian light/dark (LD) cycle (LD 3:2) in between the first and second phase assessments. They were put to bed and permitted to sleep during the 2-h dark episodes and kept awake during the 3-h light episodes in relatively dim light (~10–30 lux). The circadian clock cannot entrain to the ultradian LD cycle so it free-runs; called forced desynchrony. The phase shift of the DLMO from the first to the second phase assessment (baseline to final) was used to calculate the endogenous free-running circadian period (τ). After the second phase assessment, subjects were given access to clocks, but we controlled their LD cycle (LD 16:8) and their time in bed, in the dark. On days 7–10, they were put on an 8-h baseline sleep (dark) schedule similar to their sleep schedule at home before entering the lab. A 01:00 to 9:00 sleep schedule is shown. After the third phase assessment on days 10–11 (baseline assessment), the sleep/wake schedule, LD cycle and meal schedule were delayed (made later) by 9 h for 3 days (days 11–13). The clocks in the subjects’ bedrooms were also delayed 9 h, they were changed to Japan time; Japan is 9 time zones west of Chicago. The time line on the top shows Chicago time, and the time line on the bottom shows the corresponding time in Japan. The phase shift of the DLMO from the third to the fourth phase assessment (baseline to final) was used to determine the phase shift of the circadian clock due to the 9-h delay of zeitgebers (time cues).

**Figure 2:**
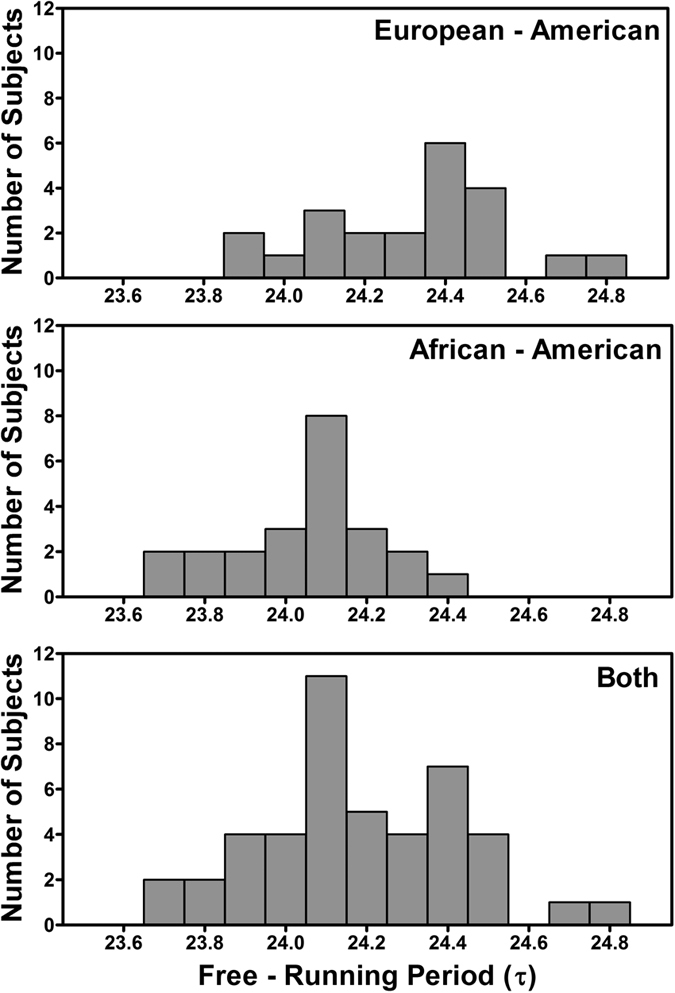
Frequency histograms of the endogenous free-running circadian periods (τ) for the European-Americans (N = 22), African-Americans (N = 23), and these subjects combined (N = 45). The free-running periods were calculated from the circadian phase assessments which were before and after the 3 days of ultradian light/dark cycles (see Fig. 1). The means ± SDs were 24.31 ± 0.24, 24.06 ± 0.19, and 24.19 ± 0.24 for the European-Americans, African-Americans and both, respectively.

**Figure 3:**
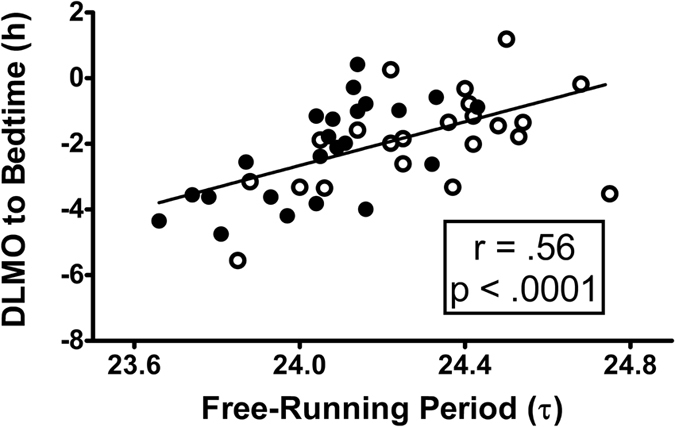
Scatter plot showing the relationship between the phase angle of entrainment of the circadian clock to the 24-h day and the free-running circadian period (τ). N = 45. Filled circles represent African-Americans (N = 23) and open circles represent European-Americans (N = 22). Phase angle of entrainment was the interval from the phase of the master, internal, circadian clock (assessed by the dim light melatonin onset, DLMO) until dark onset (bedtime = lights out). The DLMO was determined from the phase assessment after the 4 baseline days in the laboratory. A negative DLMO to bedtime interval indicates that the DLMO occurred before bedtime, which is typical. A more negative number on the y-axis indicates a longer time from the DLMO until bedtime. Subjects with shorter free-running periods were entrained to the 24-hour day with earlier circadian rhythms (earlier DLMOs) relative to dark onset (relative to bedtime), and those with longer periods entrained with later circadian rhythms relative to dark. The diagonal line represents a linear fit of the data.

**Figure 4:**
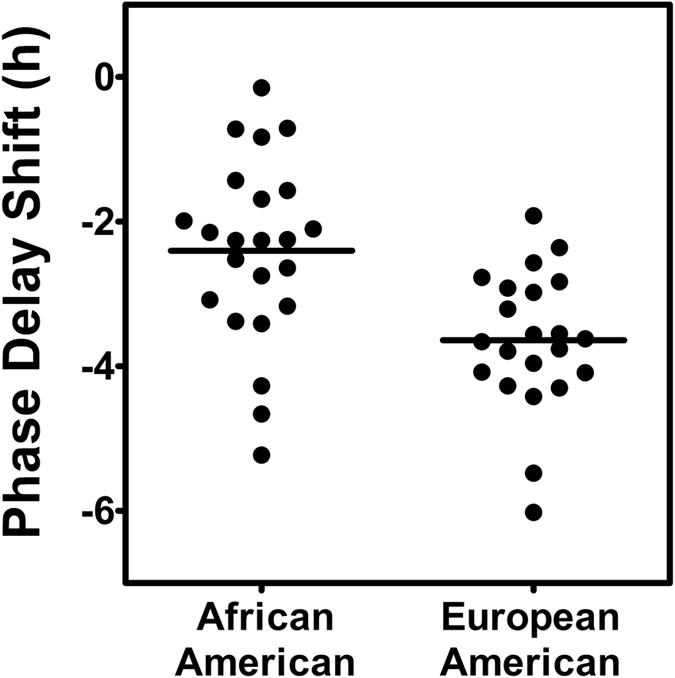
Phase shifts of the master, internal, circadian clock (assessed by the DLMO) due to the 9-hour delay of zeitgebers. Each dot represents the phase shift of an individual subject from before to after the 3 delayed sleep/dark episodes (from the third to the fourth phase assessment in Fig. 1). The horizontal lines represent the means. The phase delay (mean ± SD) was 2.4 ± 1.3 h for the 23 African-Americans and 3.6 ± 1.0 h for the 22 European-Americans (p < 0.001).

**Figure 5:**
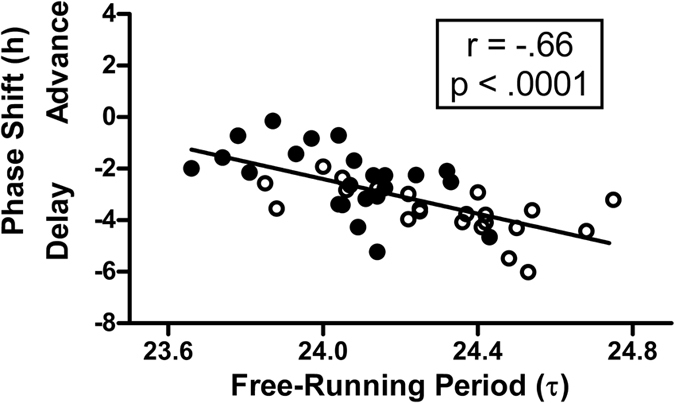
Scatter plot showing the relationship between the phase shift of the master, internal, circadian clock (assessed by the DLMO) due to the 9-hour delay of zeitgebers and the free-running circadian period (τ). N = 45. Filled circles represent African-Americans (N = 23) and open circles represent European-Americans (N = 22). Subjects with longer periods had larger phase delays. The diagonal line represents a linear fit of the data.

During the first 5 days of the protocol (see Fig. 1), subjects were not given access to phones, lap tops, clocks, watches or any device that displays clock time. Their electronic items capable of time display were locked up from when they entered the lab on day 1 until after the phase assessment on day 6. During the ultradian LD cycles (LD 3:2) subjects lived in a large, windowless, room that contained 3 beds separated by partitions. There are overhead fluorescent fixtures on dimmers which were locked to the lowest level. Light intensities were measured frequently, at each subject’s eye, at the angle of gaze, with an Extech Model EA31 digital light meter. The research assistants took readings when subjects were in all the possible various positions in the room. Analysis of 1306 measurements showed that light levels were <50 lux 99% of the time, and <30 lux 84% of the time. The median light level was 18 lux. Subjects ate and drank ad lib, but were not permitted caffeine or alcohol. Showers (1/day) were at random times. Subjects were required to remain in bed during the dark periods even if they could not sleep and were monitored by an infrared camera. While awake they sat around a large round table and ate, played games, read, watched pre-recorded movies and TV shows or engaged in other sedentary activities. After the phase assessment on day 6, subjects napped in the dark from noon to 4 pm, and were then moved to the Bedroom Suite.

The Bedroom Suite has three bedrooms, a bathroom and a control room for research assistants. This was also a windowless environment and the bedroom and hallway lights were controlled by research assistants in the control room. Subjects had their own private bedrooms and were given their cell phones and any other electronics or watches they had brought with them (laptops, tablets, etc). Baseline sleep schedules (with 8 h time in bed, in the dark) were tailored to the individual using sleep diaries kept before entering the lab to best match the subject’s natural sleep time. Each bedroom had one overhead fluorescent ceiling fixture on a dimmer switch. Each subject’s bedroom fixture was set to its maximum for the first 10 h of the 16-h wake period, dimmed to the lowest level for the last 6 h, and turned off during the 8-h sleep episodes. Research assistants took readings when subjects were in various positions in the bedrooms and hallway. Analyses of 1214 measurements showed that during the high intensity hours light levels were most often between about 30 and 300 lux, and during the low intensity hours they were between about 5 and 60 lux. The median light level was 66 lux during the high intensity time, and it was 17 lux during the low intensity time.

Meals were served at scheduled times starting when subjects were woken from the second baseline sleep episode on day 8. Breakfast was 1 h after waking, lunch was 5 h after breakfast and dinner was 6 h after lunch. In addition, subjects were allowed 2 small snacks per day of <160 calories. Caffeinated beverages and alcohol were not permitted. Each bedroom had a large wall clock set to Chicago time. When the LD cycle and the sleep schedule were delayed 9 h, the time of meals was also delayed 9 h to keep meals in the same phase relationship to the sleep schedule. The clock on the wall in each subject’s room was changed to Japan time (9 h delay), and a sign underneath the clock was changed from “Chicago” to “Japan”. Each bedroom had a bulletin board where the subject’s times for bedtime, wake time, and meals were posted. The times on these signs did not have to change when the LD cycle was delayed, because the new times were in Japan time and matched the wall clock. As during baseline, the lights were set on high for the first 10 h of the wake period and on low for the last 6 h.

The dim light melatonin onset (DLMO), our measure of the circadian phase of the internal, master, circadian clock was measured during the phase assessments (Fig. 1). Saliva samples were collected every 30 min in very dim light (<5 lux) using Salivettes (Sarstedt, Newton, NC, USA). During the phase assessments subjects sat in La-Z-Boy recliners and usually watched pre-recorded movies and TV shows. Subjects were permitted to eat and drink ad lib, but no food or drink was permitted in the 10 min before each sample. Saliva samples were centrifuged, frozen, and later sent to SolidPhase, Inc. (Portland, Maine, USA) to be radioimmunoassayed (RIA) for melatonin. Each individual’s samples were analyzed in the same batch. The sensitivity (limit of detection) of the assay was 0.9 pg/ml. Intra-assay coefficients of variation for low (daytime), medium (evening) and high (nighttime) levels were 20.1, 4.1 and 4.8% respectively. The inter-assay coefficients of variation for low, medium and high levels were 16.7, 6.6 and 8.4%, respectively.

Buccal (cheek) swabs for DNA were taken after subjects were awakened from the first baseline sleep episode on day 7, before they drank or ate anything. One hour after their wake up time they were allowed to leave the lab and go outside for 8 h. That was the only time during the entire 14-day study that they were not constantly supervised by research assistants 24 h a day. When they returned from this 8-h break they were given a urine drug screen to test for common drugs of abuse and nicotine, and were breathalyzed for alcohol. All subjects passed these screens.

### Data Analysis

Melatonin profiles were smoothed with a locally weighted least squares (LOWESS) curve set to medium, 10 points in the smoothing window (GraphPad Prism, GraphPad Software Inc., La Jolla, CA, USA). The threshold for determining the DLMO was 25% of the distance from the fitted minimum value to the fitted maximum value, i.e. minimum +25% (maximum − minimum). The second and fourth phase assessments (the two final assessments) were longer than the first and third (baseline) (Fig. 1). Therefore, the threshold from the final in each baseline/final pair was used to determine the DLMOs for that pair.

To calculate the free-running circadian period (τ) the difference between the DLMOs on the first and second phase assessment (Fig. 1) was divided by 4 (because there were 4 days between these DLMOs) and then added to 24 (when the DLMO delayed), or subtracted from 24 (when the DLMO advanced). For example if the DLMO on the first assessment was 21:00 and the DLMO on the second was 22:00, then the difference is 1.0 h. We divide this difference by 4 which equals 0.25 h. The circadian period is thus 24.25 h. If the DLMO on the first assessment was 23:00 and the DLMO on the second was 22:00, then the circadian period would be 23.75 h.

To calculate the phase shift of the circadian clock due to the 9-h delay of zeitgebers the DLMO on the fourth phase assessment was subtracted from the DLMO on the third phase assessment (Fig. 1). Thus, if the DLMO on the third assessment was 21:00 and the DLMO on the fourth was 23:00 then the phase shift would be −2.0 h. By convention delays are indicated with a negative number.

We used t-tests to examine differences between African-Americans and European- Americans in variables such as the free-running period and phase shift, and for a sex difference in circadian period among the European-Americans. Pearson correlation coefficients were used to test for associations among the main outcomes measures. All reported tests are based on 2-tailed probabilities. Results are presented as means ± SD unless otherwise indicated. GraphPad Prism was used for data analysis.

## Additional Information

**How to cite this article**: Eastman, C. I. *et al.* Circadian rhythms of European and African-Americans after a large delay of sleep as in jet lag and night work. *Sci. Rep.* **6**, 36716; doi: 10.1038/srep36716 (2016).

**Publisher’s note**: Springer Nature remains neutral with regard to jurisdictional claims in published maps and institutional affiliations.

## Figures and Tables

**Table 1:** Subject Demographics.

	African American	European American
N	23	22
Sex	10 Men 13 Women	12 Men 10 Women
Age Range	21 to 44	18 to 41
Age (Mean ± SD)	32 ± 7.1	29 ± 6.5
MSF^a^ (Mean ± SD)	4.8 ± 1.2	5.2 ± 1.1
MEQ^b^		
Score (Mean ± SD)	56.5 ± 8.5	51.3 ± 8.8*
# of M-Types	12	5
# of N-Types	10	15
# of E-Types	1	2
BMI (kg/m^2^) (Mean ± SD)	25.0 ± 3.9	23.5 ± 3.4
Genetic Ancestry^c^ (Mean ± SD)		
% European	13.1 ± 9.9	86.1 ± 9.7
% Sub-Saharan African	78.7 ± 10.3	5.9 ± 7.6
% East Asian	3.4 ± 6.1	3.6 ± 5.5
% Indigenous American	4.7 ± 6.5	4.2 ± 6.0

*Significant difference between African-Americans and European-Americans (p < 0.05 by 2-tailed t-test). Higher scores indicate more morningness.

There were no differences between African-Americans and European-Americans in age, MSF or BMI.

^a^Mid Sleep on Free Days from Munich Circadian Type Questionnaire (MCTQ).

^b^Morningness-Eveningness Questionnaire (MEQ) score and number of Morning, Neither and Evening Types.

^c^Biogeographical ancestry estimates based on ancestry informative markers.

**Table 2:** Circadian period (τ), baseline circadian phase, sleep schedule, phase angle of entrainment, and phase shifts to 9-h delay of zeitgebers. Mean ± SD.

	African American	European American
N	23	22
Free-Running Period (τ) (h)	24.06 ± 0.19	24.31 ± 0.24*
Baseline Circadian Phase^a^ (h:min)	21:37 ± 2:05	22:35 ± 1:53
Bedtime^b^ (h:min)	23:52 ± 1:08	0:27 ± 1:11
Waketime^b^ (h:min)	7:52	8:27
Phase Angle of Entrainment^c^ (h)	−2.3 ± 1.5	−1.9 ± 1.5
Phase Delay^d^ (h)	−2.4 ± 1.3	−3.6 ± 1.0*

^*^Significant difference between African-Americans and European-Americans (p < 0.001 by 2-tailed t-test).

^a^The dim light melatonin onset (DLMO) from a phase assessment after the four 8-h baseline sleep episodes in the lab.

^b^Scheduled baseline bedtime (dark onset). Scheduled wake time (light onset) was always 8 h later.

^c^The phase angle of entrainment of the circadian clock to the 24-h zeitgebers during baseline, calculated as the interval from the baseline DLMO to baseline bedtime (dark onset). Negative numbers indicate that the DLMO occurred before bedtime.

^d^Phase shift of the circadian clock (assessed by the DLMO) after the 9 h delay of zeitgebers.
